# A Novel Antimicrobial Peptide Scyreprocin From Mud Crab *Scylla paramamosain* Showing Potent Antifungal and Anti-biofilm Activity

**DOI:** 10.3389/fmicb.2020.01589

**Published:** 2020-07-24

**Authors:** Ying Yang, Fangyi Chen, Hui-Yun Chen, Hui Peng, Hua Hao, Ke-Jian Wang

**Affiliations:** ^1^State Key Laboratory of Marine Environmental Science, College of Ocean and Earth Sciences, Xiamen University, Xiamen, China; ^2^State-Province Joint Engineering Laboratory of Marine Bioproducts and Technology, College of Ocean and Earth Sciences, Xiamen University, Xiamen, China

**Keywords:** *Scylla paramamosain*, antimicrobial peptide, scyreprocin, antifungal activity, anti-biofilm activity

## Abstract

Natural antimicrobial peptides (AMPs) are potential antibiotic alternatives. Marine crustaceans are thought to generate more powerful and various AMPs to protect themselves from infections caused by pathogenic microorganisms in their complex aquatic habitat, thus becoming one of the most promising sources of AMPs or other bioactive substances. In the study, a novel protein was identified as an interacting partner of male-specific AMP SCY2 in *Scylla paramamosain* and named scyreprocin. The recombinant product of scyreprocin (rScyreprocin) was successfully expressed in *Escherichia coli*. rScyreprocin exerted potent, broad-spectrum antifungal, antibacterial, and anti-biofilm activity (minimum inhibitory concentrations from 0.5 to 32 μM) through differential modes of action, including disruption of cell membrane integrity and induction of cell apoptosis, and has rapid bactericidal (in 0.5–2 h) and fungicidal (in 8–10 h) kinetics. In addition to its fungicidal activity against planktonic fungi, rScyreprocin also prevented the adhesion of fungal cells, inhibited biofilm formation, and eradicated the mature biofilms. Moreover, rScyreprocin showed a profound inhibitory effect on spore germination of *Aspergillus* spp. (minimum inhibitory concentrations from 4 to 8 μM). This peptide was not cytotoxic to murine and mammalian cells and could increase the survival rate of *Oryzias melastigma* under the challenge of *Vibrio harveyi*. Taken together, the novel AMP scyreprocin would be a promising alternative to antibiotics used in aquaculture and medicine.

## Introduction

Since antibiotics revolutionized medicine in the early 20th century (Gualerzi et al., 2013), antibiotic medications have been widely used in not only clinical treatments and prevention of microbial infections but also feedstuff (Brown et al., 2017). However, inappropriate treatments and excessive use of antibiotics have led to the constant emergence of antibiotic-resistant microorganisms (Santos and Ramos, 2018; Ghosh et al., 2020). In recent years, these antibiotic-resistant pathogens have caused an increasing number of infections, such as pneumonia (Chalmers et al., 2013), septicemia (Ferguson and Rhoads, 2009), and urinary tract infections (Kahlmeter and ECO.SENS, 2003), which have been considered as a great threat to global public health. Moreover, healthcare-associated infections (HAIs), especially medical device HAIs caused by biofilms, are of great concern due to the enormous threat they posed and the corresponding financial burden (Busscher et al., 2012; Kurtz et al., 2012; Zmistowski et al., 2013), which make them a public health challenge to be addressed. Thus, there is an urgent need to control the abuse and overuse of antibiotics worldwide and explore effective antibiotic alternatives. In the past decades, studies focusing on antimicrobial peptides (AMPs) have rekindled the possibility of seeking more promising anti-pathogenic drugs in addition to traditional antibiotics (Hancock and Sahl, 2006; Haney et al., 2017).

Antimicrobial peptides are short peptides with broad-spectrum antimicrobial activities. As an important component of innate immunity, AMPs have been found in almost all life forms and play important roles in defending against pathogen invasions (Jenssen et al., 2006) and assisting both innate and adaptive immune functions by activating complement and chemoattract cells (Brogden et al., 2016). This non-specific mechanism of action is highly effective, allowing AMPs to possess multiple activities against a variety of microorganisms (Cushnie et al., 2016). Alternatively, some AMPs translocate into microbial cells to interact or bind with intracellular molecules, then inhibit key cellular processes (DNA, RNA, protein and cell wall synthesis, etc.) (Brogden, 2005; Wilmes et al., 2011), and activate autolysin and/or hinder the activity of certain enzymes. Also, some AMPs have been proved to induce apoptosis and trigger cell death of target microorganisms (Epand and Epand, 2010; Cho and Lee, 2011; Hwang et al., 2011). It is generally believed that AMPs might eliminate different microbial species via distinct or combined action modes, indicating that different AMPs might have different antimicrobial mechanisms. The diverse physiochemical properties and cellular targets exhibited in different AMPs would facilitate the selection of suitable alternatives for the targeted antibiotics. Besides, antibiotic-resistant bacteria exhibit collateral sensitivity to AMPs, but relatively rare cross-resistance is observed (Lam et al., 2016; Lázár et al., 2018), which makes them ideal alternatives to antibiotics. Moreover, AMPs have also been considered as potential candidates against biofilms formed by multidrug-resistant microbes (Dobson et al., 2011; Batoni et al., 2016; Chung and Khanum, 2017). Natural AMPs and designed AMP derivatives have broad application prospects in the treatments of chronic diseases caused by multidrug-resistant microbes and prevention of microbial colonization of medical implants and therapies on virus-bacterium coinfection (Melvin et al., 2016; Sánchez-Gómez and Martínez-de-Tejada, 2017; Lázár et al., 2018). However, some natural AMPs have weak antibacterial activity *in vitro*, whereas others are difficult to express through genetic engineering or chemical synthesis, thus limiting their application. Therefore, accelerating the discovery of new AMPs and the development of effective AMP expression systems or synthetic technologies will facilitate the mechanism study and potential applications of AMPs.

Crustaceans (such as mud crab) are invertebrates that could only rely on the innate immune system to fight infections. To date, dozens of AMPs have been isolated from hemocytes of different species of crustaceans, and they have been proved to be effective in eliminating pathogenic microorganisms (Fredrick and Ravichandran, 2012). Besides, several AMPs are identified in the reproductive system of vertebrates or invertebrates, such as defensins (Com et al., 2003), histone H1-like protein (Nam et al., 2012), and LEAP-2 (Michailidis, 2010). In our previous studies, scygonadin (GenBank: AY864802) (Huang et al., 2006) and its homologous protein SCY2 (GenBank: DQ872630) (Qiao et al., 2016) were found in the male gonads of mud crab *Scylla paramamosain* and display inhibitory effect on several pathogens. Both peptides could be transferred to female spermatheca through the mating process, so we speculate that these peptides may play protective roles in successfully maintaining fertilization (Xu et al., 2011a, b). However, the physiological functions of scygonadin and SCY2 have not yet been fully elucidated.

In the study, we performed a yeast-two hybrid (Y2H) assay to screen for potential SCY2-interacting proteins, thereby identifying for the first time a previously uncharacterized protein and naming it scyreprocin. To characterize this new peptide, we first expressed scyreprocin in *Escherichia coli* and obtained its product (rScyreprocin). The antimicrobial activity of rScyreprocin was determined *in vitro*. After minimum inhibitory concentration (MIC) assay, scanning electron microscope (SEM), transmission electron microscope (TEM), flow cytometry, and confocal laser scanning microscopy assays were used to further investigate the antimicrobial features on various microorganisms *in vitro*. Also, the *in vivo* protective efficiency of rScyreprocin in marine medaka *Oryzias melastigma* under the challenge of the aquatic pathogen *Vibrio harveyi* was further evaluated.

## Materials and Methods

### Animals and Strains

Mud crab (*Scylla paramamosain*) were obtained from Xiamen aquatic products market: male crabs (bodyweight 300 ± 10 g) were used for Y2H complementary DNA (cDNA) library construction and co-immunoprecipitation (co-IP) assay, and male crabs (bodyweight 100 ± 10 g) were used for co-IP assay. Marine medaka (*Oryzias melastigma*) was farmed in the State Key Laboratory of Marine Environmental Science. Tissue samples were dissected using sterile tools, flash-frozen in liquid nitrogen, and transferred to −80°C for storage. All animal experiments were carried out in strict accordance with the guidelines of Xiamen University. The microbes used in this study were listed in Appendix 1.

### Bait Strain Construction for Yeast-Two Hybrid Assay

The gene encoding SCY2 mature peptide was amplified from the cDNA of male mud crab gonads and cloned in frame with the GAL4 DNA-binding domain (BD) of the yeast expression vector pGBKT7 (Clontech, CA, United States) to construct the bait plasmid (pGBKT7-SCY2). Expression of a BD-SCY2 fusion protein in the yeast strain Y2HGold (Clontech, CA, United States) was then confirmed by Western blotting using antibodies against BD and SCY2.

### Construction of *Scylla paramamosain* Male Gonad Complementary DNA Library and Yeast-Two Hybrid Screening

Total RNA was extracted from the freshly sampled sexual gonads of male mud crabs, concentration and integrity of which was checked by Agilent 2100. Mixed equal amount of RNA from each gonad part for cDNA library construction using Make Your Own Mate & Plate^TM^ Library System (Clontech). The Size-fractionation step recommended in manufacturer’s protocol was skipped because SCY2 might interact with low-molecular-weight peptide. Titer and diversity of the cDNA library were determined as described in the manufacturer’s protocol. The Y2H screening was performed three times using the Matchmaker^TM^ Gold Yeast Two-Hybrid System (Clontech). Prey plasmids from the positive clones were then rescued and sequenced.

### Cloning, Expression, Purification, and Analysis of Recombinant Proteins

Recombinant SCY2 (rSCY2) was cloned, expressed, and purified as previously described (Peng et al., 2011). The open reading frame of scyreprocin was constructed into expression vectors pET28a(+) and pGEX4T2 and transformed into *E. coli* BL21 (DE3) and further tested for recombinant protein expression (primer sequences were listed in Supplementary Table 1). The rScyreprocin expressed by *E. coli* BL21/pET28a-scyreprocin was purified through HisTrap^TM^ FF crude (GE Healthcare, United States) on an ÄKTA Pure system (GE Healthcare, United Kingdom) following the standard protocol. Glutathione *S*-transferase (GST)-tagged scyreprocin fusion protein expressed by *E. coli* BL21/pGEX4T2-scyreprocin was purified using glutathione magnetic agarose beads (Thermo Fisher, United States).

Purified proteins were dialyzed and concentrated, and the storage buffer was 50-mM sodium phosphate buffer (NaPB; pH 8.0). Protein concentration was determined by Bradford assay. Purified proteins were submitted to sodium dodecyl sulfate–polyacrylamide gel electrophoresis (SDS-PAGE) analysis, Western blotting, and mass spectrometry identification. All recombinant proteins were stored at −80°C.

### Polyclonal Antibody Preparation

Full-length amino acid sequence of scyreprocin was subjected to Optimum Antigen^TM^ design tool for antigen site prediction. Synthesis of selected antigen sites (CSGNKGKESKDPKVK), preparation, and quality inspection of the antibody were performed by GenScript (NJ, CHN). SCY2 antibody was prepared as previously described (Qiao et al., 2016). The specificity of SCY2 and scyreprocin antibodies were analyzed following the standard Western blotting procedure.

### Co-immunoprecipitation Assay

Seminal plasma extracts of sexually mature (bodyweight 300 ± 10 g) and immature (bodyweight 100 ± 10 g) male crabs were collected and adjusted to a final concentration of 1 mg ml^–1^ and pre-treated with Protein-A/G beads (GenScript, CHN) to remove non-specific binding. Protein-A/G beads and SCY2 antibody (1:1,000) were co-incubated overnight with seminal plasma extracts with gentle rotation at 4°C. Beads were collected and washed with ice-cold phosphate-buffered saline (PBS, pH 7.4) to remove unbound fractions and subjected to SDS-PAGE analysis, silver staining, and Western blotting with scyreprocin antibody (1:1,000).

### Mammalian Two-Hybrid Assay

The mammalian two-hybrid (M2H) assay was performed on human cervical carcinoma cell (HeLa cells, kindly provided by Stem Cell Bank, Chinese Academy of Sciences) using the Checkmate^TM^ M2H system (Promega, United States) in accordance with the manufacturer’s instructions. Briefly, gene fragments encoding SCY2 mature peptide and scyreprocin were cloned in frame with pACT vector and pBIND vector (primer sequences were listed in Supplementary Table 1), respectively. HeLa cells were maintained in minimal essential medium (Invitrogen, United States) supplemented with 10% fetal bovine serum (FBS; Gibco). Cells were plated at ∼5.0 × 10^4^ cells well^–1^ on a 48-well cell culture plate (Thermo Fisher). Vector combinations, including (1) pACT/pBIND/pG5luc, (2) pACT-SCY2/pBIND/pG5luc, (3) pACT/pBIND-scyreprocin/pG5luc, and (4) pACT-SCY2/pBIND-scyreprocin/pG5luc, were transiently transfected into HeLa cells using the ViaFect^TM^ Transfection Reagent (Promega) according to the manufacturer’s instructions. Cells were harvested and lysed in passive lysis buffer (Promega) at 48-h post-transfection. Reporter activities were measured using the Dual-Luciferase Reporter Assay System (Promega) on a GloMax 20/20 luminometer (Promega). Experiments were performed in triplicate.

### Glutathione *S*-Transferase Pull-Down

GST-tagged scyreprocin (4 μg ml^–1^) and rSCY2 (4 μg ml^–1^) were co-incubated overnight with glutathione magnetic agarose beads (Thermo Fisher) at 4°C. Beads were washed intensively with 50-mM PBS (pH 7.4), followed by SDS-PAGE analysis.

### Far-Overlay Western Blotting

For the far-overlay Western blotting assay, purified GST-tagged scyreprocin and GST (as control) were transferred to a polyvinylidene difluoride membrane. The membrane was blocked with 5% skim milk in Tris-buffered saline Tween-20 (10-mM Tris-HCl, 150-mM NaCl, and 0.05% [v/v] Tween-20) and incubated overnight with rSCY2 (25 nM) at 4°C. The blots were incubated with SCY2 antibody (1:2,000) for 1 h, washed with Tris-buffered saline Tween-20, incubated with horseradish peroxidase (HRP)-conjugated goat anti-mouse immunoglobulin G (IgG) secondary antibody (1:5,000) for another 1 h, and detected with an ECL Western blotting substrate.

### Surface Plasmon Resonance Assay

All the following experiments were carried out with a Biacore T200 and CM5 sensor chip (GE Healthcare). To evaluate the interaction between rScyreprocin and rSCY2, rSCY2 was immobilized on flow cell 2 (flow cell 1 was used as a blank reference) of CM5 sensor chip to a total signal increase of ∼1,000 resonance units. The multiple cycle kinetic assay was performed at 25°C with a flow rate of 30 μl min^–1^ in running 4-(2-hydroxyethyl)-1-piperazineethanesulfonic acid-buffered saline (GE Healthcare). After baseline equilibration, rScyreprocin was diluted in 4-(2-hydroxyethyl)-1-piperazineethanesulfonic acid-buffered saline (12.5, 25, 50, 100, 200, and 400 nM), injected, and analyzed. Data were recorded at a rate of 10 Hz during 120-s association and 120-s dissociation phases.

### Antimicrobial Activity of rScyreprocin

A batch of microbial strains, including common microbes and pathogenic microbes, was used to evaluate antimicrobial activity of rScyreprocin, rSCY2, and isomolar concentration mixture of rScyreprocin and rSCY2 (rScyreprocin/rSCY2). MIC and minimum bactericidal concentration (MBC) were determined in triplicate on separate occasions following the liquid growth inhibition assay protocol described before (Shan et al., 2016).

### Time-Killing Kinetic Assessment

Two bacterial (*Pseudomonas stuzeri* and *Micrococcus lysoleikticus*) and two fungal strains (*Candida albicans* and *Cryptococcus neoformans*) were subjected for time-killing studies following prior description (Wang et al., 2009; Shan et al., 2016). Recombinant proteins (rScyreprocin and rScyreprocin/rSCY2) were incubated with bacteria at concentrations of 4 and 8 μM and incubated with fungi at concentrations of 32 and 64 μM. Cultures were sampled and plated at various time points. Plates were incubated at 37°C for 18–24 h, and the total viable count (TVC) was estimated. The assays were performed in triplicate. Killing efficiency was determined by using the survival rate of colony-forming unit (% CFU) calculated as follows: % CFU = recovered CFU/initial CFU × 100%, where initial CFU means TVC at 0 min and recovered CFU means TVC at different sampling points.

### Biofilm Inhibition Assays

*Candida albicans* and *C. neoformans* were cultured in yeast extract peptone dextrose (YPD) to their logarithmic growth phase and harvested by gentle centrifugation. To assess the inhibition effect of the recombinant proteins on microbial adhesion, fungal cells were resuspended and adjusted to 2 × 10^6^ CFU ml^–1^ in YPD containing various concentrations of rScyreprocin, rSCY2, and rScyreprocin/rSCY2 (0, 2, 4, and 8 μM), respectively, and then added 100 μl to each well of a 96-well plate. Plates were incubated at 37°C for 90 min to allow cell adhesion. Planktonic cells were removed, and the wells were gently washed three times with 50-mM NaPB (pH 7.4). The adhered microbes were evaluated by crystal violet staining as previously described (Berditsch et al., 2015). To determine inhibitory effect of the recombinant proteins on biofilm formation, fungi were resuspended in YPD and incubated for 90 min at 37°C. Planktonic microbes were removed, and the attached fungi were incubated with YPD containing different concentrations of proteins. After incubation for 24 and 48 h, biofilm mass was evaluated as described earlier. To evaluate the eradication effect of the recombinant proteins on biofilms, *C. albicans* and *C. neoformans* were cultured in YPD for 24 h to form biofilms. Various dilutions of proteins (prepared in YPD) were then added to the biofilms and incubated at 37°C. Biofilm mass was determined as described earlier. The assays were carried out in triplicate.

### Propidium Iodide Uptake Assay

To investigate changes in microbial membrane integrity after rScyreprocin treatment, the influx of propidium iodide (PI) was measured using a modified protocol (Lin et al., 2013). Briefly, *P. stutzeri* and *C. albicans* were resuspended (approximately 10^8^ CFU ml^–1^) and incubated with NaPB (solution control) or rScyreprocin (8 μM) for 30 min. PI was added to the culture (final concentration = 100 μg ml^–1^) and incubated at room temperature for 5 min in the dark. Fluorescent images were captured with a multiphoton laser scanning microscope (Zeiss Lsm 780 NLO, Germany). The fluorescent intensity of PI was quantified using Carl Zeiss ZEN 2011 software (blue edition).

### Microbial Genomic DNA-Binding Assay

To evaluate the binding capacity of rScyreprocin and microbial genome, gel retardation experiment was performed. In total, 300 ng of the bacterial genomic DNA (*Staphylococcus aureus*, *P. stutzeri*, and *C. albicans*) were mixed with a serial dilution of rScyreprocin (0–96 μM, prepared in deionized water) and incubated at room temperature for 30 min (total volume = 10 μl). Reaction mixture was electrophoresed in a 0.8% agarose gel and imaged by a gel imaging system (FluorChem^TM^ FC3 system, ProteinSimple, United States).

### Binding Properties of rScyreprocin to Bacterial Cell Surface Components

To determine the binding properties of rScyreprocin with lipopolysaccharides (LPS B5, Sigma, United States), lipoteichoic acid (LTA, L2515, Sigma, United States), and peptidoglycan (PGN from *Bacillus subtilis*, Sigma, United States), a modified ELISA assay was performed following prior description (Shan et al., 2016). Briefly, a flat bottom 96-well ELISA plate was coated with LPS, LTA, or PGN (3 μg), blocked with 5% (w/v) BSA, and incubated with serial dilutions of rScyreprocin, rSCY2, and rSCY2/rScyreprocin (0–24 μM, 100 μl well^–1^), respectively. After washing with PBS for three times, the plates were incubated with 100-μl mixture containing scyreprocin antibody (1:2,000) and SCY2 antibody (1:1,000) for 2 h before incubating with mixture of HRP-labeled goat anti-rabbit IgG and HRP-labeled goat anti-mouse IgG (1:1,000) for 1 h. After the colorimetric reaction, the absorbance at 450 nM was measured using a microplate reader (TECAN GENios). The experiments were carried out in triplicate, and Scatchard plot analysis was applied to assess the results. Apparent dissociation constant (*K*_d_) was generated by Scatchard analysis. Interaction strengths were defined as “strong” (*K*_d_ < 100 nM), “moderate” (100 nM to 10 μM), and “weak” (>10 μM) (Watson et al., 1976). The chitin-binding property of the recombinant proteins was determined following prior description (Shan et al., 2016).

### Scanning Electron Microscope and Transmission Electron Microscope Examinations of Microbial Morphology

For electron microscopic observation, nutrient broth, marine broth 2216E, YPD, and potato dextrose water were used as the growth medium for bacteria, vibrio, fungi, and spores, respectively. Sterile tube containing 1 ml of growth medium only and 1 ml of growth medium supplemented with rScyreprocin were inoculated with approximately 5 × 10^5^ CFU of microbes. After incubation for 30 min, microbial cells were collected and fixed with pre-cooled 2.5% (w/v) glutaraldehyde at 4°C for 2 h. For SEM observation, microbes were dehydrated and gold-coated following prior descriptions (Lin et al., 2013) before observed by a Zeiss Supra^TM^ 55 scanning electron microscope. For TEM observation, microbes were subjected for ultrathin sections and negative stained following standard protocols (Chen et al., 2003) before further observed by a transmission electron microscopy (FEI Tecnai G2 F20).

### Localization of rScyreprocin in Microbes

rScyreprocin localization studies were carried out as previously described (Shan et al., 2016). After a 10-min incubation at 30°C, microbes were fixed with 4% (w/v) paraformaldehyde for 30 min, subsequently immobilized on a poly-L-lysine-coated glass slide at 4°C for 1 h. The microbial cells were permeabilized with 0.1% (v/v) Triton X-100 (prepared in PBS, pH 7.4) and blocked with 10% (v/v) normal goat serum (NGS) for 1 h at room temperature. Subsequently, specimens were incubated with scyreprocin antibody (1:500, diluted in 1% [v/v] NGS) overnight at 4°C followed by incubation in dark with Dylight 650 conjugated goat anti-rabbit IgG (1:1,000, diluted in 1% [v/v] NGS) for 1 h at room temperature. 4′, 6-Diamidino-2-phenylindole was applied for chromatin staining. Samples were observed and imaged with a multiphoton laser scanning microscope (Zeiss Lsm 780 NLO, Germany). The fluorescent intensity of rScyreprocin was quantified using Carl Zeiss ZEN 2011 software (blue edition).

### Apoptotic Assay

To investigate whether rScyreprocin could induce apoptosis of *C. albicans*, fungal cells were incubated with culture media supplemented with various concentrations of rScyreprocin (0, 2, 4, and 8 μM) at 28°C for 1 h. Samples were subjected for 4′, 6-diamidino-2-phenylindole staining and observed with a multiphoton laser scanning microscope (Zeiss Lsm 780 NLO). For flow cytometry analysis, *C. albicans* was incubated with various concentrations of rScyreprocin (1 and 4 μM) for various time periods (0.5, 1, and 2 h) were collected and labeled using Annexin V-APC as recommended by the manufacturer (BD Bioscience). Ratio of annexin V-positive *C. albicans* cells in each group was then analyzed by an Attune NxT flow cytometer (Thermo Fisher).

### Cytotoxicity Test

Mouse hepatic cells (AML12) and human hepatic cells (L02) were kindly provided by Stem Cell Bank, Chinese Academy of Sciences. AML12 cells were maintained in Dulbecco’s Modified Eagle Medium/Nutrient Mixture F-12 (Invitrogen, United States) supplemented with 10% FBS (Gibco), human insulin (10 μg ml^–1^), human transferrin (5.5 μg ml^–1^), sodium selenite (5 ng ml^–1^), and dexamethasone (40 ng ml^–1^). L02 cells were maintained in RPMI-1640 media supplemented with 10% FBS. Cells were plated at ∼2.0 × 10^4^ cells well^–1^ on a 96-well cell culture plate (Thermo Fisher) and incubated overnight at 37°C in a humidified atmosphere with 5% CO_2_. Cells were incubated with culture media supplemented with NaPB (solution control) or rScyreprocin (0.5, 1, 2, 4, 8, and 16 μM). After incubation for 24 h, cell viability was assessed using a CellTiter 96^®^ AQueous Kit (Promega). Experiments were carried out in triplicate.

### Evaluation of the *in vivo* Activity of rScyreprocin on *Vibrio harveyi*-Infected *Oryzias melastigma*

To investigate *in vivo* protective effect of rScyreprocin, marine medaka *O. melastigma* and its pathogenic bacteria *V. harveyi* were chosen as study subjects. Marine medaka were temporary reared for 3 days before experiment. *V. harveyi* were cultured in marine broth 2216E to their logarithmic growth phase and harvested by centrifugation at 3,000 *g* for 10 min at room temperature. Cells were resuspended in fish saline and injected into medaka at concentrations ranged from 8.3 × 10^5^ to 1.3 × 10^7^ CFU fish^–1^ (fishes injected with fish saline were set up as injection controls; each group contained 2 tanks, 10 fish tank^–1^). Fish mortality was recorded at 24-h post-injection (hpi), and half lethal dose (LD50) at 24 hpi was then determined. Similar as prior description, marine medakas were injected with *V. harveyi* at LD50, and various dosages of rScyreprocin, rSCY2 (0, 1, 2, 4, and 8 μg fish^–1^, prepared in fish saline), and rScyreprocin/rSCY2 (i.e., 1 μg fish^–1^ rScyreprocin/rSCY2: 1 μg rScyreprocin and 1 μg rSCY2 per fish) were injected at 2-h post-*V. harveyi* injection (fish injected with fish saline followed by another fish saline injection were set up as operating controls; fish injected with fish saline followed by various concentration of protein dilutions were served as protein controls; fish injected with *V. harveyi* followed by fish saline were set up as *V. harveyi*-infection controls; each group contained 2 tanks, 10 fish tank^–1^). Fish mortality in each group was monitored and recorded for 24 h; survival rate was calculated.

### Statistical Analysis

For the biofilm formation assessment data, statistical analyses were performed by two-way analysis of variance (ANOVA) following a Bonferroni post-test. M2H results were analyzed by one-way ANOVA, repeated measurement. Statistical analyses were performed using GraphPad Prism 6.0 Software (GraphPad Software Inc., CA, United States), with a confidence level of 95% being considered statistically significant.

## Results

### Scyreprocin Is a Novel SCY2-Interacting Partner

**FIGURE 1:**
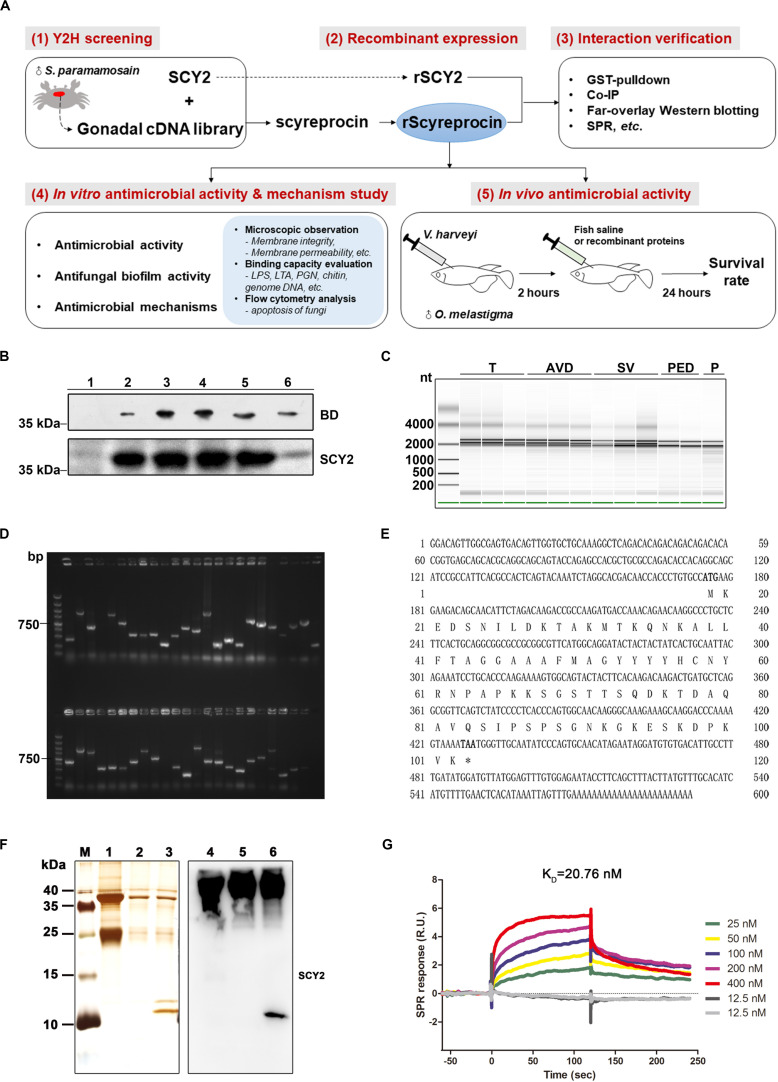
Identification and verification of the SCY2-interacting protein. **(A)** Schematic diagram of the research route of rScyreprocin. **(B)** Expression of the fusion protein BD-SCY2 of the constructed bait strain was detected by Western blotting. **(C)** Total RNA extracted from different gonadal segments of sexually mature male *S. paramamosain* was examined for integrity by Agilent 2000. **(D)** Gel electrophoresis analyzation on diversity of the constructed cDNA library; DNA marker: DL2000. **(E)** Full-length cDNA and the deduced amino acid sequences of scyreprocin. **(F)** Co-immunoprecipitation analysis of the *in vivo* scyreprocin–SCY2 interaction in seminal plasma of sexually immature (lanes 2, 5) and mature (lanes 3, 6) crabs. Lanes 1 and 4, rabbit IgG controls. **(G)** Binding kinetic of rScyreprocin and rSCY2 assessed by surface plasmon resonance technology.

The schematic of this paper was summarized in Figure 1A. To search for potential SCY2-interacting partners in *S. paramamosain*, a yeast two-hybrid (Y2H) assay was performed. The mature peptide of SCY2 was used as the bait sequence (Figure 1B). RNAs extracted from gonads of sexually matured male crabs was used to construct a high-quality cDNA library for Y2H assay (Figure 1C). Evaluation of the constructed cDNA library showed good diversity and was qualified to apply for further screening (Figure 1D). The quality evaluation of Y2H assay was listed in Supplementary Table 2. Approximately 5.32 × 10^4^ diploids were screened, and 612 positive colonies were identified. The prey plasmids were rescued from the positive clones and retransformed into Y187 and mated with the bait strain to confirm the interaction (Supplementary Figure 1A). Sequencing of the positive clones showed the same result. Data analysis showed that there was no similar nucleotide or amino acid sequence in existing online databases, indicating that it is an uncharacterized protein. We named it scyreprocin (GenBank: MH488960).

The full-length cDNA sequence of scyreprocin was confirmed from the testicular RNA of *S. paramamosain*. This new gene is 591 bp, including a 255-bp open reading frame (Figure 1E). Scyreprocin contains 84 residues, of which lysine (15%), alanin (11%), and serine (9%) are the main amino acid types, with a total hydrophobic ratio of 27%. The calculated mass of scyreprocin is 9.107 kDa with an estimated isoelectric point (pI) of 9.6. The total net charge of scyreprocin was +7, suggesting that it is a cationic protein. Recombinant GST-tagged scyreprocin, His-tagged scyreprocin (rScyreprocin), and SCY2 (rSCY2) were expressed and purified (Supplementary Figures 2A–C). The interaction between SCY2 and scyreprocin was further verified by co-IP assay (Figure 1F), M2H assay (Supplementary Figure 1B), far-overlay Western blotting (Supplementary Figure 1C), and GST pull-down (Supplementary Figure 1D). The surface plasmon resonance assay indicated that the calculated equilibrium dissociation constant (*K*_D_) of the rScyreprocin–rSCY2 interaction was 20.76 nM (Figure 1G).

### rScyreprocin Exerts Potent Broad-Spectrum Antimicrobial Activity

The antimicrobial and bactericidal activities (shown in MIC and MBC) of rScyreprocin were examined (Table 1). The data showed that rScyreprocin exerted broad-spectrum antibacterial and antifungal activity. Most tested Gram-negative and Gram-positive bacteria were susceptible to rScyreprocin (MICs from <0.5–4 μM and MBCs from <0.5–8 μM). The anti-vibrio activity of rScyreprocin was also investigated. Common aquatic pathogenic *Vibrio fluvialis* and *V. harveyi* were susceptible to low concentrations of rScyreprocin (MICs from 1 to 4 μM), whereas *Vibrio alginolyticus* and *Vibrio parahaemolyticus* were less susceptible (MICs from 4 to 16 μM).

**TABLE 1:** Antimicrobial activity of rScyreprocin, rSCY2, and rScyreprocin/rSCY2.

Microorganisms	CGMCC No.^a^	rScyreprocin		rSCY2	rScyreprocin/rSCY2
		
		MIC (μM)^b^	MBC (μM)^b^	MIC (μM)	MIC (μM)
**Gram-negative bacteria**					
*Pseudomonas fluorescens*	1.0032	<0.5	1–2	>50	1.5–3
*Pseudomonas stutzeri*	1.1803	0.5–1	1–2	>50	1.5–3
*Shigella flexneri*	1.1868	<0.5	>15	>50	1.5–3
*Escherichia coli*	1.2389	2–4	>15	>50	3–6
*Escherichia coli MC1061*	–	2–4	4-8	>50	3–6
*Aeromonas hydrophila*	1.2017	>15	>15	>50	>24
*Vibrio fluvialis*	1.1609	1–2	2–4	>50	6–12
*Vibrio harveyi*	1.1593	2–4	8–16	>50	6–12
*Vibrio alginolyticus*	1.1833	4–8	16–32	>50	6–12
*Vibrio parahaemolyticus*	1.1615	8–16	>32	>50	>24
**Gram-positive bacteria**					
*Micrococcus luteus*	1.634	<0.5	<0.5	25–50	<1.5
*Micrococcus lysoleikticus*	1.0634	<0.5	1–2	12.5–25	3–6
*Staphylococcus aureus*	1.363	<0.5	2–4	>50	<1.5
*Corynebacterium glutamicum*	1.1886	2–4	2–4	25–50	3–6
*Bacillus subtilis*	1.108	1–2	4–8	12.5–25	3–6
**Fungi**					
*Cryptococcus neoformans*	2.1563	1–2	8–16	>50	8–16
*Candida albicans*	2.2411	2–4	16–32	>50	6–12
*Candida krusei*	2.1857	8–16	>32	>50	16–32
*Candida parapsilosis*	2.1846	16–32	>32	>50	>24
*Candida tropicalis*	2.1975	16–32	>32	>50	>24
*Pichia pastoris*	2.2238	4–8	>30	>50	16–32
*Fusarium graminearum*	3.349	8–16	>32	>50	16–32
*Fusarium solani*	3.584	8–16	>32	>50	16–32
*Fusarium oxysporum*	3.6785	16–32	>32	>50	16–32
*Aspergillus niger*	3.0316	4–8	>32	>50	32–64
*Aspergillus ochraceus*	3.583	4–8	>32	>50	8–16
*Aspergillus fumigatus*	3.5835	4–8	>32	>50	16–32
*Neurospora crassa*	3.1604	16–32	>32	>50	16–32

*^*a*^China general microbiological culture collection number; ^*b*^MIC and MBC were presented as an interval [A]–[B]: [A] was the highest concentration tested with visible microbial growth, whereas [B] was determined as the lowest concentration without visible microbial growth (*n* = 3).*

Common pathogenic yeasts, including *C. neoformans* and *Candida* spp. (MICs from 1 to 32 μM), were susceptible to rScyreprocin, and rScyreprocin showed fungicidal effect against *C. neoformans* and *C. albicans* (Table 1). In addition, rScyreprocin inhibited the spore germination of several tested molds, especially against that of *Aspergillus* spp. (MICs from 4 to 8 μM) (Supplementary Figure 3). The synthesized scyreprocin showed no antimicrobial activity against all tested microbes, and neither the combination of rSCY2 and rScyreprocin showed synergistic antimicrobial effect (Table 1).

The antimicrobial activity of three synthetic scyreprocin fragments were measured. The results showed that all the scyreprocin fragments had weak or no inhibitory effect against most of the microbes that inhibited by rScyreprocin (Supplementary Table 3). However, scyreprocin [20–39] and scyreprocin [40–84] could inhibit the growth of *Aeromonas hydrophila* (Supplementary Table 3), which was not susceptible to rScyreprocin treatment.

### rScyreprocin Exerts Rapid Bactericidal and Fungicidal Kinetics

**FIGURE 2:**
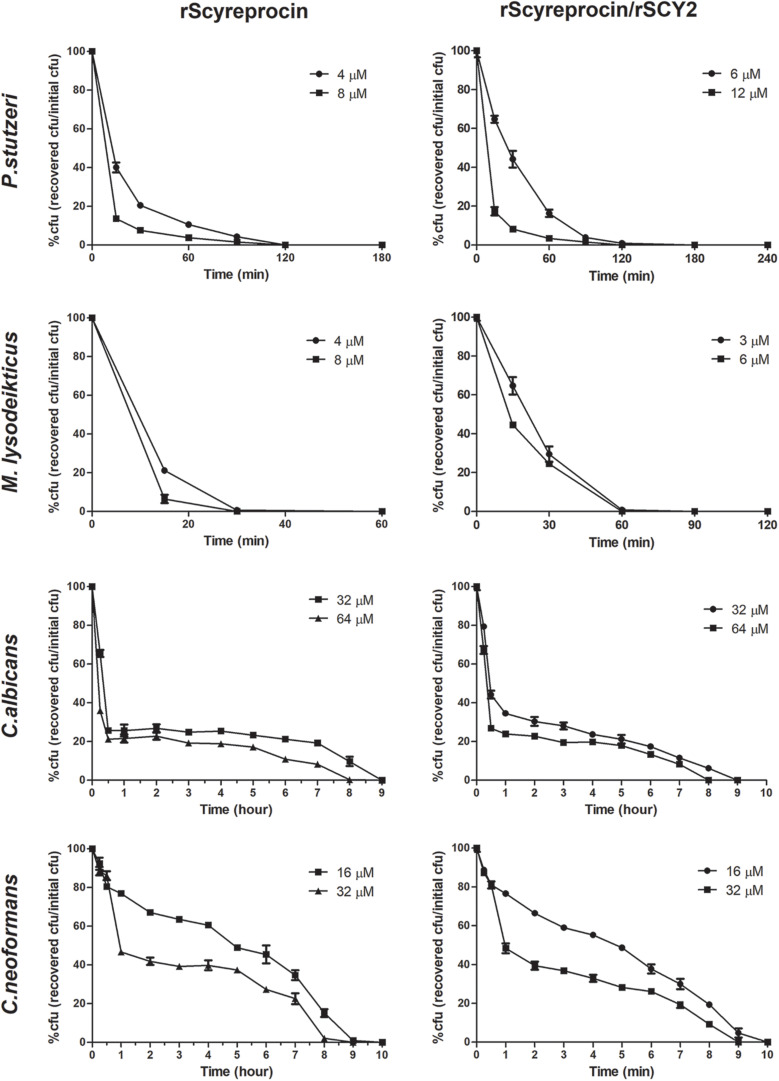
Time-killing curves of *Pseudomonas stutzeri*, *Micrococcus lysoleikticus*, *Candida albicans*, and *Cryptococcus neoformans* treated with rScyreprocin and rScyreprocin/rSCY2. The percentage of CFU is defined relative to the CFU obtained in the control (100% CFU at 0 min). Data represent means ± SEMs of three independent determinations (*n* = 3).

Killing kinetics of rScyreprocin on *Micrococcus lysodeikticus*, *P. stutzeri*, *C. albicans*, and *C. neoformans* were evaluated. Time-killing kinetic assay showed that when rScyreprocin was incubated with *M. lysodeikticus* and *P. stutzeri* at a concentration of 8 μM, all bacteria could be killed after incubation for 30 and 180 min, respectively. However, rScyreprocin (32 μM) eliminated 50% of the fungal cells in 1 h, but it took another 7–8 h to kill the remaining half (Figure 2). The combination of rScyreprocin and rSCY2 could not enhance the bactericidal and fungicidal activities.

### rScyreprocin Has Multiple Antimicrobial Mechanisms

After treatment with rScyreprocin for 30 min, compared with the control groups, the microbes surface was clearly rougher, with deep craters, membrane rupture, and even cytoplasmic leakage. The same phenomenon was observed in spores and mycelium treated with rScyreprocin (Supplementary Figure 4). The TEM images of *P. stutzeri* and *E. coli* showed clear formation of bacterial outer membrane vesicles, enlargement of intermembrane space, rupture of inner membrane, and leakage of cytoplasm in a concentration-dependent manner (Supplementary Figure 5).

**FIGURE 3:**
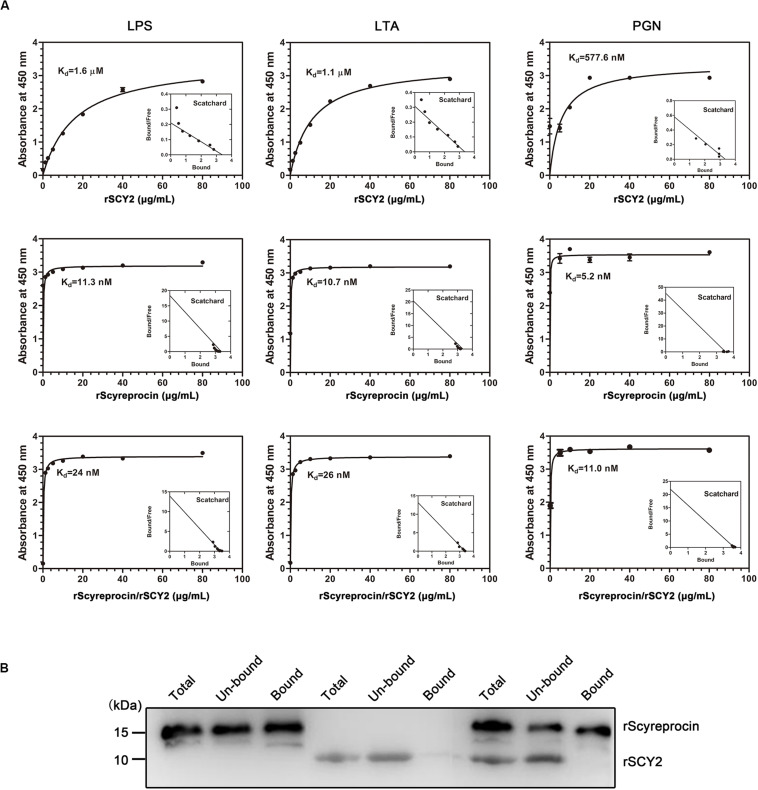
Binding property of rScyreprocin, rSCY2, and rScyreprocin/rSCY2 to microbial associated molecules. **(A)** Binding affinity of rScyreprocin, rSCY2, and rScyreprocin/rSCY2 to LPS, LTA, and PGN assessed by ELISA, respectively. Data represent as means ± SEMs from three independent experiments (*n* = 3). **(B)** Chitin binding capacity of rScyreprocin, rSCY2, and rScyreprocin/rSCY2. The total-, unbound-, and bound-fraction were analyzed by SDS-PAGE followed by Western blotting using anti-scyreprocin (1:1,000) and anti-SCY2 (1:1,000) mixture as primary antibody.

**FIGURE 4:**
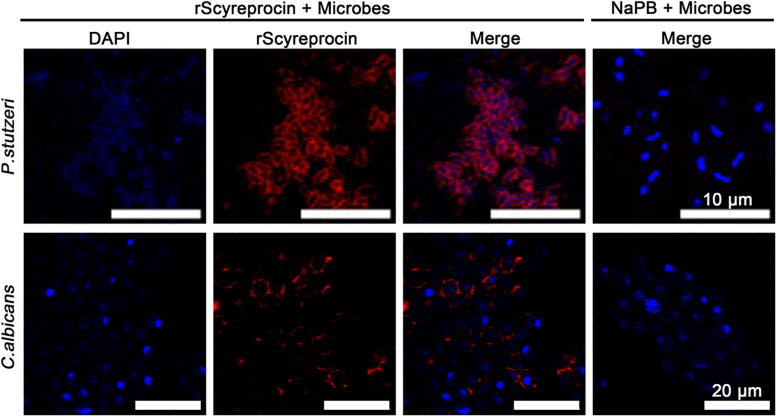
Localization of rScyreprocin in *Pseudomonas stutzeri* and *Candida albicans*. Exponential phase microbial cells were resuspended in culture media supplemented with NaPB (control) or rScyreprocin (4 μM) for 10 min. Samples were submitted for immunofluorescence analysis and observed by a confocal laser scanning microscopy. For the microbes treated with NaPB, only merged images are shown.

**FIGURE 5:**
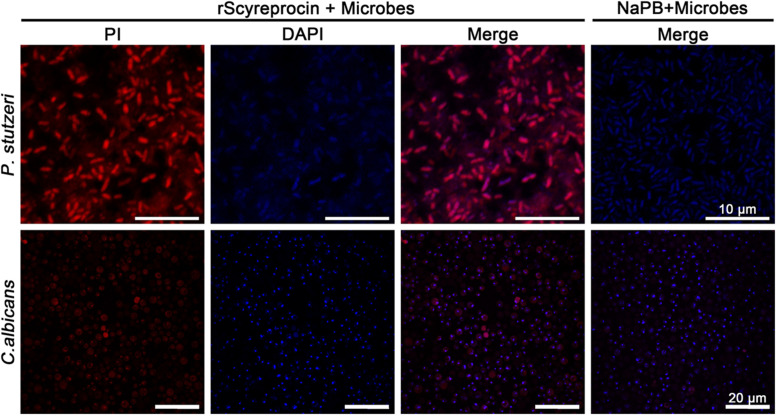
Effect of rScyreprocin on the membrane integrity of *Pseudomonas stutzeri* and *Candida albicans*. Exponential phase microbial cells of *P. stutzeri* and *C. albicans* were resuspended in culture media supplemented with NaPB (control) or rScyreprocin (8 μM) for 30 min. The samples were supplemented with PI and observed using a confocal laser scanning microscopy. For the microbes treated with NaPB, only merged images are shown.

To elucidate the underlying antimicrobial mechanism of rScyreprocin, we first assessed the binding properties of rScyreprocin to different microbial surface molecules. Scatchard plot analysis showed that rScyreprocin had high binding affinity to LPS, LTA, and PGN with apparent dissociation constants (*K*_d_) of 11.3, 10.7, and 5.2 nM, respectively (Figure 3A). In the chitin-binding assay, rScyreprocin was recovered in the bound fractions, indicating its interaction with chitin (Figure 3B). Recombinant SCY2 could also bind with LPS, LTA, PGN, and chitin; however, rScyreprocin/rSCY2 showed no significant synergistic binding affinity to the tested microbe associated molecules (Figure 3). Immunofluorescence assay revealed that rScyreprocin exerted its antimicrobial activity through targeting bacterial membrane (Figure 4). PI-uptake assay demonstrated that almost all the tested microbial cells were labeled with PI fluorescence after a 10-min incubation with rScyreprocin, whereas no fluorescence signal was visualized in the control group (Figure 5). The earlier discussed experimental results suggested that rScyreprocin might first attach to the microbial surface, damage the microbial membrane or induce membrane permeability change, and then, fulfill its antimicrobial function.

**FIGURE 6:**
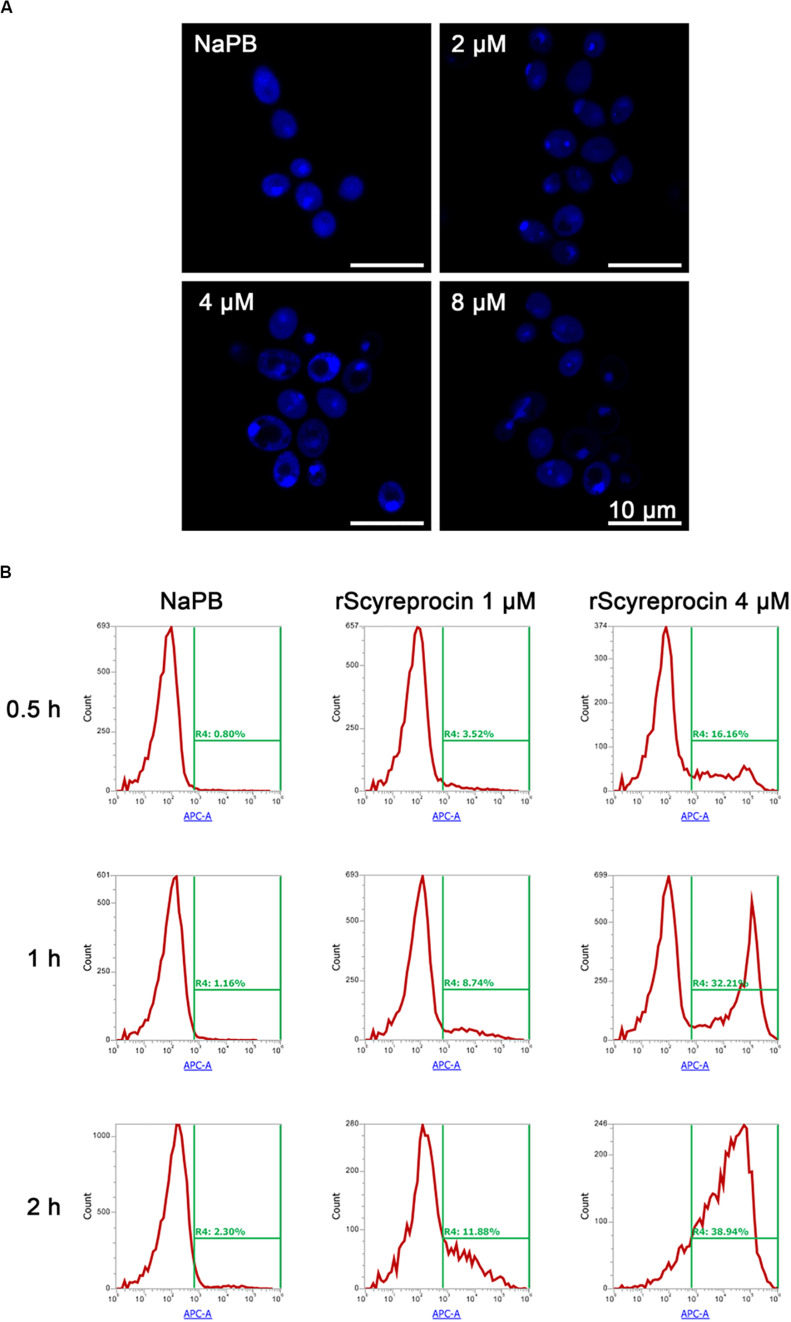
Apoptotic effect of rScyreprocin on *Candida albicans*. **(A)** Exponential phase microbial cells of *C. albicans* were resuspended in culture media supplemented with NaPB (control) or rScyreprocin for 1 h at 28°C. Samples were then applied for 4′, 6-diamidino-2-phenylindole staining and observed for nucleus changes by a confocal scanning laser microscopy. **(B)** Exponential phase microbial cells of *C. albicans* were resuspended in culture media supplemented with NaPB (control) or rScyreprocin (1 and 4 μM) at 28°C for various time period (0.5, 1, and 2 h). Samples were then processed for flow cytometry analysis of apoptosis using Annexin V-APC.

*Candida albicans* treated with rScyreprocin showed a clear nuclear morphological change and chromatin condensation (Figure 6A). Furthermore, rScyreprocin treatment induced cell apoptosis of *C. albicans* in a concentration–time-dependent manner (Figure 6B). Besides, the rScyreprocin showed no positive binding property with microbial genome DNA (gDNA) (Supplementary Figure 6). From the earlier discussed, rScyreprocin targeted and disrupted the integrity of bacterial membranes rather than the bacterial gDNA to kill bacteria, while exerting its effect on fungi by both membrane destruction and apoptosis induction.

### rScyreprocin Prevents the Adhesion of Planktonic Yeasts, Inhibits Biofilm Formation, and Eradicates Mature Fungal Biofilms

**FIGURE 7:**
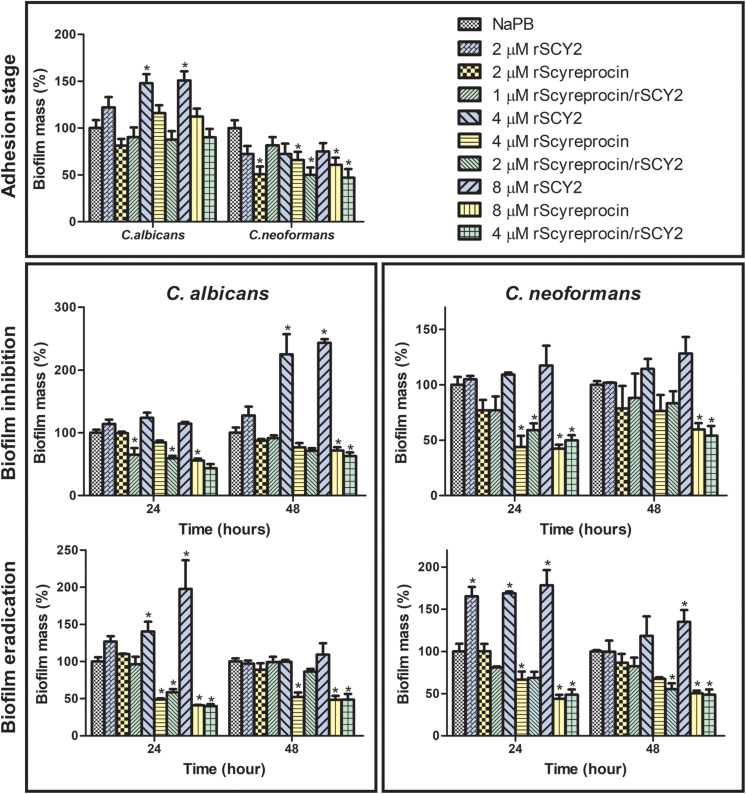
Effect of rScyreprocin and rSCY2 on fungal biofilms formed by *Cryptococcus neoformans* and *Candida albicans*. Yeasts or the corresponding biofilms from *C. neoformans* and *C. albicans* were kept untreated or were treated with various concentrations of rScyreprocin, rSCY2, and rScyreprocin/rSCY2 and then processed to crystal violet staining for evaluation of biofilm formation levels. Data represent means ± SEMs from three independent experiments (*n* = 3), representing the relative percentage of biofilm mass (control groups were normalized to 100%). ^∗^*P* < 0.05 versus control (two-way ANOVA, Bonferroni post-test).

Since planktonic yeasts were susceptible to rScyreprocin, we tested whether this susceptibility extended to biofilms. Results showed that rScyreprocin not only inhibited the biofilm formation but also eradicated mature biofilms of *C. albicans* and *C. neoformans* (Figure 7). *C. albicans* and *C. neoformans* cells were allowed to adhere to the poly-lysine coated wells of 96-well plates and treated with rScyreprocin at different time points to evaluate its potential effect during adhesion stage, biofilm formation, and on mature biofilm. As shown in Figure 7, rScyreprocin reduced the adhesion of *C. neoformans* (2, 4, and 8 μM). The treatment of rScyreprocin was able to suppress biofilm formation and eradicate mature biofilm of both *C. albicans* and *C. neoformans* in a concentration-dependent manner. In Figure 7, the average value of the biofilm mass (%) of *C. neoformans* during the adhesion stage after rSCY2 treatment was lower than that of the NaPB group, but there was no statistical difference. Higher concentrations of rSCY2 might be able to prevent fungi cell adhesion; however, the exact mechanism needs further investigation. In combination of rSCY2, better biofilm inhibition and eradication effects were achieved with lower dose of rScyreprocin.

### rScyreprocin Shows No Cytotoxicity

**FIGURE 8:**
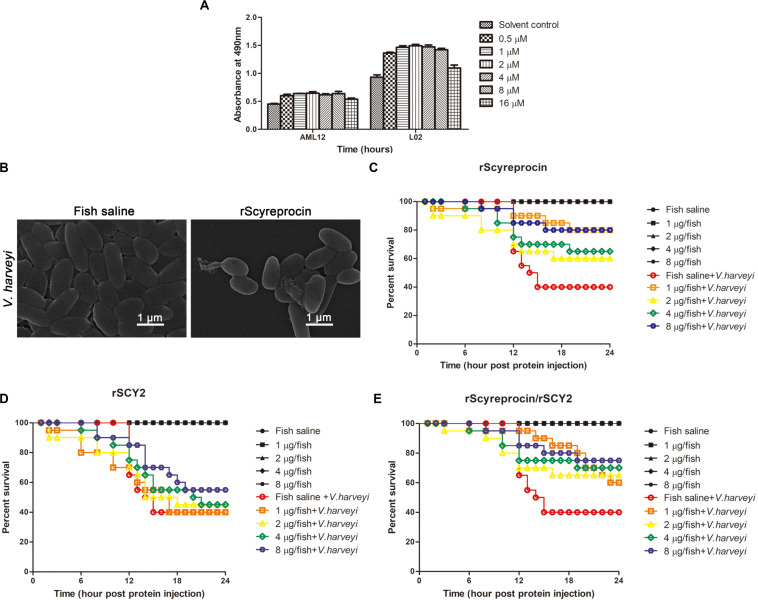
*In vivo* protective effect of rScyreprocin, rSCY2, and rScyreprocin/rSCY2 on *Vibrio harveyi*-infected *Oryzias melastigma*. **(A)** Cytotoxicity effect of rScyreprocin on mammalian cells. Murine and human hepatic cells, AML12 and L02, were incubated with culture media supplemented with various concentrations of rScyreprocin, and cell viability was determined by MTS method. Data represent the means ± SEMs from three independent experiments. **(B)** Scanning electron microscopy images showing the effect of rScyreprocin on *V. harveyi* observed. **(C–E)** Fish were challenged with *V. harveyi* at semi-lethal dose, and injections of rScyreprocin **(C)**, rSCY2 **(D)**, and rScyreprocin/rSCY2 **(E)** at various dosages were given at 2-h post-bacterial challenge (*n* = 20 for each group). The survival curve of each experimental group was analyzed using the Kaplan–Meier log-rank test.

To determine whether rScyreprocin could be safely used in *in vivo* experiments, the cytotoxicity of rScyreprocin was analyzed using AML12 and L02 cell lines (Figure 8A). rScyreprocin showed no cytotoxicity. In contrast, the cell viability of both AML12 and L02 cells was improved.

### rScyreprocin Improves the Survival of *Vibrio harveyi*-Infected *Oryzias melastigma*

rScyreprocin could inhibit the growth of aquatic pathogenic *V. harveyi* through a membrane disruptive mechanism (Figure 8B). To assess the antimicrobial activity of rScyreprocin *in vivo*, marine medaka (*O. melastigma*) was challenged with a semi-lethal dose of *V. harveyi* (LD50, Supplementary Figure 7). After 2 h of bacterial challenge, different amounts of rScyreprocin were injected into the fish. The survival rate of the control group decreased to 40% at 24-h post-bacterial injection, whereas the protein injection control groups (without bacterial challenge) showed a 100% survival rate. The survival rate of the experimental groups receiving rScyreprocin injection was increased to 80% (Figure 8C). Thus, rScyreprocin significantly improved the survival of *V. harveyi*-infected marine medaka but did not show a negative effect. The potential protective effects of rSCY2 and rScyreprocin/rSCY2 were also evaluated. Although not shown to be toxic to marine medaka, rSCY2 alone exerted only a weak protective effect against *V. harveyi* infection (Figure 8D). When used in combination with rSCY2, the dosage of rScyreprocin can be reduced, and at the same time, the survival rate of fish can be guaranteed, indicating they have a synergistic protective effect *in vivo* (Figure 8E).

## Discussion

To date, more than 3,000 peptides have been registered in the Antimicrobial Peptide Database (APD), and most of the identified and well-studied AMPs are from terrestrial animals (Wang et al., 2015). The ocean, occupying 70% of our planet, nurtures more than half of the world’s biological species. However, only a small proportion of the reported AMPs comes from marine resources (Wang et al., 2015). To adapt to the extreme conditions of their habitats (such as temperature, salinity, pressure, pollutants, abundant pathogenic microbes, and numerous viruses), marine animals may produce marine natural products (MNPs) with remarkable diversity in structure compared with terrestrial counterparts. Therefore, identification of AMPs from marine animals, especially marine crustaceans, has attracted the attention of many researchers. The AMPs identified in crustacean [e.g., tachyplesins (Muta et al., 1990), crustins (Liu et al., 2015), and penaeidins (Destoumieux et al., 2000)] are proved to possess potent antimicrobial activity, indicating that crustaceans are unparalleled resources of bioactive substances. Mud crabs molt multiple times during their lifespan and undergo a long fertilization process to reproduce. Thus, the pathogenic microbes in the aquatic environment pose a serious threat to their survival and successful reproduction. Because mud crab is lack of adaptive immune system, the AMPs in hemolymph and reproduction system are believed to be the key components to protect mud crab from infections. We have identified several AMPs from hemolymph (SpHyastatin, etc.) and gonads (scygonadin, SCY2, etc.) of the mud crab (Huang et al., 2006; Qiao et al., 2016; Shan et al., 2016), and the present study is a continuation of our ongoing works on characterizing new AMPs from mud crab. In this study, a novel AMP, scyreprocin, was identified as an interacting partner of SCY2 from the reproductive system of male *S. paramamosain*. The recombinant products of this peptide (rScyreprocin) exert potent inhibitory effect against bacteria (both Gram-negative and Gram-positive bacteria), fungi, fungal biofilms, and mold spore germination *in vitro*. rScyreprocin exerts its function via a variety of action modes, including disruption of membrane integrity (bacteria, fungi, mold spores, and hyphae) and induction of cell apoptosis (fungi). rScyreprocin did not exhibit toxicity and showed favorable *in vivo* antimicrobial activity, implying its broad application prospects in agriculture, aquaculture, and clinical treatments.

One antimicrobial mechanism of AMPs is to destroy the integrity of microbial membrane and ultimately leads to the release of the cytoplasm (Li et al., 2017). Seawater carries numerous bacteria, including pathogenic ones, and almost all pathogenic microbes of *S. paramamosain* are Gram-negative bacteria, such as *Vibrio parahaemolyticus*, *V. alginolyticus*, and *Pseudomonas putida* (Valerie et al., 1993). Among the previously identified mud crab AMPs, only a few AMPs, such as *Sp*PR-AMP1 (Imjongjirak et al., 2017) and SpHyastatin (Shan et al., 2016), showed anti-*Vibrio* activity. In this study, rScyreprocin showed profound antimicrobial activity against most of the tested bacterial strains, including mud crab pathogens *P. putida*, *V. parahaemolyticus*, *V. harveyi*, etc. The present study revealed that rScyreprocin exerted a rapid bactericidal effect by quickly binding to molecules on a bacterial surface in a short period (10 min), affecting membrane permeability, damaging membrane integrity, and inducing cytoplasmic leakage within 30 min. During the mating process (which lasts for 10–12 h), the male gametes of *S. paramamosain* might come into contact with seawater and become a window for infections, which endangers the success of reproduction. Therefore, when gametes were infected by pathogenic bacteria during mating, scyreprocin might immediately attach to the bacterial surface, rupture the cell membrane, and lyse the bacterial cells. Although scyreprocin was identified as an interacting partner of SCY2, in our *in vitro* study, these two AMPs showed no synergistic antimicrobial activity as well as LPS-binding affinity, and the addition of rSCY2 seemed to attenuate the activity of rScyreprocin. It has been reported that the site of curcumin in myeloid differentiation protein 2 overlaps the binding site of LPS; therefore, curcumin could inhibit the binding of myeloid differentiation protein 2 to LPS (Jerala, 2007). Some other previous studies also suggested that certain LPS-binding peptides (e.g., HPep1, HNP-1, and gramicidin) could inhibit the binding of LPS to LPS-binding protein by binding to different target sites on LPS (Hancock and Scott, 2000; Ju et al., 2011). Therefore, we speculated that rSCY2 attenuated antimicrobial activity of rScyreprocin in part by affecting the binding of rScyreprocin to LPS.

Among all the animal-derived AMPs registered in the APD (∼2,344), majority of them (∼2,069) are antibacterial, and only ∼916 of them are antifungal, and most of the antifungal peptides are from terrestrial sources (Wang et al., 2015). APD screening showed that antifungal peptides in marine animals are mainly identified in shrimps (15), crabs (14), green sea turtles (3), and sea urchins (2). The reported marine-derived AMPs were tested against only a limited number of fungi (mainly *C. albicans*) to determine their antifungal activity. In addition to *C. albicans*, rScyreprocin was tested against several fungal strains that caused systematic candida infection (*C. tropicalis*), meningitis (*C. neoformans*), sepsis and wound-tissue infections (*Candida parapsilosis*), and nosocomial infection (especially in intensive care unit) in immune-compromised patients and those with hematological malignancies (*Candida krusei*). Our study revealed that rScyreprocin has a potent antifungal effect against *Cryptococcus* spp. and *Candida* spp. *in vitro*, with MIC values that range from 2 to 32 μM, and the fungicidal activity against *C. albicans* and *C. neoformans* (MBC values that range from 8 to 32 μM). Most clinical fungal diseases are caused by candida. Azoles, polyenes, echinocandins, and 5-fluorocytosine are the preferred antifungal drugs (Butts and Krysan, 2012). These drugs kill fungi by interacting with certain targets in fungal cells. For example, azoles inhibit the synthesis of ergosterol in fungal cell membrane by inhibiting fungal cytochrome P450-dependent enzymes, thus producing toxic sterols that can accumulate in cells, affect the fluidity and permeability of cell membranes, and achieve fungicidal effects (Yoshida, 1988). Echinocandins inhibit activity of glucan synthase, causing the imperfection of cell wall, imbalance of osmotic pressure, and finally lysis of fungal cells (Denning, 2003). 5-Fluorocytosine enters the fungal cell, transferred into 5-fluorouracil, and integrated into RNA, therefore interfering with proteins, inhibiting DNA synthesis, and nucleus division (Waldorf and Polak, 1983). Polyenes could directly bind to ergosterol, damage cell membrane, and affect membrane permeability and metabolism, thereby inhibiting the growth of fungi (Kitajima et al., 1976). Although the antifungal drugs are effective, their possible adverse effects and toxicity limit their application (Laniado-Laborín and Cabrales-Vargas, 2009). Moreover, due to the abuse of antifungal drugs, the global emergence of antifungal drug resistance poses a real threat to human health (Fredrick and Ravichandran, 2012; Fisher et al., 2018).

Fungi generate drug resistance by enhancing the expression of drug efflux pump, overexpressing or mutating the drug target enzymes, reducing the expression of drug targets, or forming biofilms (Pfaller, 2012). Unlike the common antifungal drugs, rScyreprocin kills fungi by disrupting cell structure and inducing cell apoptosis. Although there is no conventional chitin-BD in scyreprocin, it located on the fungal surface by direct interaction with chitin and further destroyed the integrity of cell structure within a short time (30 min). This non-specific membrane disruptive action mode endows rScyreprocin with quick fungicidal ability and makes it difficult for fungi to develop corresponding drug resistance. Additionally, rScyreprocin had an alternative way to ensure its fungicidal activity by inducing cell apoptosis. This antifungal feature of rScyreprocin was similar to that of miltefosine (Spadari et al., 2018). After rScyreprocin treatment, increased membrane permeability and chromatin condensation of *C. albicans* were observed. According to our test, it would be mentioned that rScyreprocin did not bind to the gDNA of *C. albicans* and its possible molecular basis of inducing cell apoptosis will be further investigated.

One way for fungi to develop resistance is through the formation of biofilms. Fungal biofilm is a strong structure that is resistant to chemical drugs, host immune responses, and environmental stresses (Santos et al., 2017). Fungal biofilm are responsible for chronic diseases and HAIs, endanger human health, and impose a huge economic burden throughout the world (Busscher et al., 2012). Unlike their planktonic counterparts, microbes in biofilm exhibit unique clinical features, such as persistence and stronger virulence, which make them difficult to resolve (Chandra et al., 2001). Most antifungal drugs only effectively inhibit planktonic fungal cells during the exponential growth phase but have little effect on biofilms. Currently used antibiotics, including gentamicin (Scott and Higham, 2003), rifampicin (Sanz-Ruiz et al., 2018), and clindamycin (Neut et al., 2005; Ooi et al., 2017), have been applied on devices to cope with medical device HAIs, but the consequent emergence of antibiotic-resistance bacteria raise more questions (Thornes et al., 2002). Other antimicrobial agents, such as metals (Antonelli et al., 2012) and bacteriophages (Lu and Koeris, 2011), have been tested for their ability to remove biofilm. It has been shown that currently available antibiotics damage the host microbiota, potentially leading to reinfection of opportunistic pathogens capable of forming biofilms (Kostakioti et al., 2013). Natural compounds, because of their potential probiotic effects, are beneficial for profitable colonizers, while in turn, inhibiting pathogenic microbes, thus attracting more attention (Jagani et al., 2009; Carraro et al., 2014). In addition to the fungicidal effect, rScyreprocin also exhibits inhibitory activity at different stages of biofilm formation. The ability of rScyreprocin to simultaneously kill planktonic fungi and destroy biofilms is a favorable characteristic for its future clinical application as an antifungal compound. When used in combination with rSCY2, although rSCY2 alone did not show any inhibitory effect on the tested fungus, the mixture further reduced fungal adhesion, inhibited biofilm formation, and eradicated mature biofilms with a lower dose of the rScyreprocin. The combination of rScyreprocin and rSCY2 might be a promising biocompatible coating that could prevent fungal medical device-associated infection or a promising therapeutic agent to prevent biofilm-related diseases. However, the inhibitory effect of rScyreprocin on fungal biofilm was not observed on the bacterial biofilms in our study. In our preliminary experiments, rScyreprocin showed no inhibitory effect on the formation of *S. aureus* biofilm (data not shown), and the antibacterial biofilm activity of rScyreprocin against more pathogenic bacterial strains needs further investigation. In recent years, peptide synthesis technology allows us to design and obtain antibiofilm peptides with desired activity and low cytotoxicity, which might be the next-generation antimicrobials with promising applications (César de la et al., 2016). Natural AMPs have subtle properties that may have been selected by evolution, so they can be used as ideal primitive templates for drug design and synthesis (Marcelo et al., 2019). rScyreprocin conferred good antibiofilm activity and no cytotoxicity. Further study on the antibiofilm mechanism of rScyreprocin and identification of the corresponding functional domains will help to evaluate its application prospect and its value as a drug design template.

Molds produce mycotoxins, and this destructive compounds can cause quality degradation of raw material for feed and industry and even endanger the lives of animals (Hussein and Brasel, 2001). rScyreprocin not only inhibited the growth of bacteria and yeast but also showed inhibitory effects on the spore germination of several common molds such as *Aspergillus* spp., *Fusarium* spp., and *Neurospora crassa*. This finding strongly indicated its application prospect as a natural antiseptic in food and forage industry. Last but not least, rScyreprocin, either used alone or in combination with rSCY2, has shown good antimicrobial activity *in vivo*. Future studies on whether rScyreprocin could eliminate pathogens by directly acting or modulating the immune system like other AMPs (Björn et al., 2012; Lin et al., 2012; Shan et al., 2016; Ma et al., 2017) is beneficial to its application.

To date, many MNPs have been found to be promising drug candidates to cure drug-resistant bacterial infections, and several MNPs or their derivatives have been entered clinical trials or are approved for use (Cheung et al., 2015). Despite the various obstacles, the discovery of new antibacterial and antifungal drugs from the ocean and the application of MNPs in clinical treatment are increasing. In summary, this study has demonstrated the bioactivity and the related mechanism of scyreprocin, a newly discovered SCY2-interacting AMP in mud crab *S. paramamosain*. Recombinant scyreprocin exerted potent antimicrobial activity against a broad spectrum of pathogenic microorganisms. Moreover, rScyreprocin exhibited profound antifungal and inhibitory effect on fungal biofilm formation. Recombinant scyreprocin showed no cytotoxicity on both murine and mammalian cells and could significantly improve fish survival under bacterial infection. This peptide can effectively kill microbes via multiple modes of action, including membrane-disruptive mechanism and induction of cell apoptosis. The discovery and characterization of this bio-friendly AMP with potent broad-spectrum antimicrobial activity would provide promising clues for its future development as a novel antimicrobial drug. Additionally, the detailed work in this study indicated that mud crab is a valuable resource for the discovery of unique bioactive substances. Therefore, it may be a beneficial attempt to discover new AMPs using a comprehensive genomic approach.

## Data Availability Statement

The datasets generated for this study can be found in the GenBank Accession Number: MH488960.

## Ethics Statement

The animal study was reviewed and approved by the Laboratory Animal Management and Ethics Committee of Xiamen University.

## Author Contributions

K-JW and YY conceived and designed as well as analyzed the experiments. YY performed all the experiments and wrote the manuscript. FC assisted the writing and contributed to the preparation of the figures. H-YC provided the technical assistance in laser scanning confocal microscope imaging. HP and HH provided the technical assistance in the expression of recombinant protein. K-JW also contributed all of reagents, materials, and analysis tools and wrote the manuscript. All the authors reviewed the results and approved the final version of the manuscript.

## Conflict of Interest

The authors declare that the research was conducted in the absence of any commercial or financial relationships that could be construed as a potential conflict of interest.
