# Supplementary material for: A Novel Antimicrobial Peptide Scyreprocin From Mud Crab Scylla paramamosain Showing Potent Antifungal and Anti-biofilm Activity
Source: Front Microbiol. 2020 Jul 24;11:1589. doi: 10.3389/fmicb.2020.01589 (PMC7396596; doi:10.3389/fmicb.2020.01589)
Supplement: Supplementary file 1 [file Data_Sheet_1.docx]

**Supplementary Information**


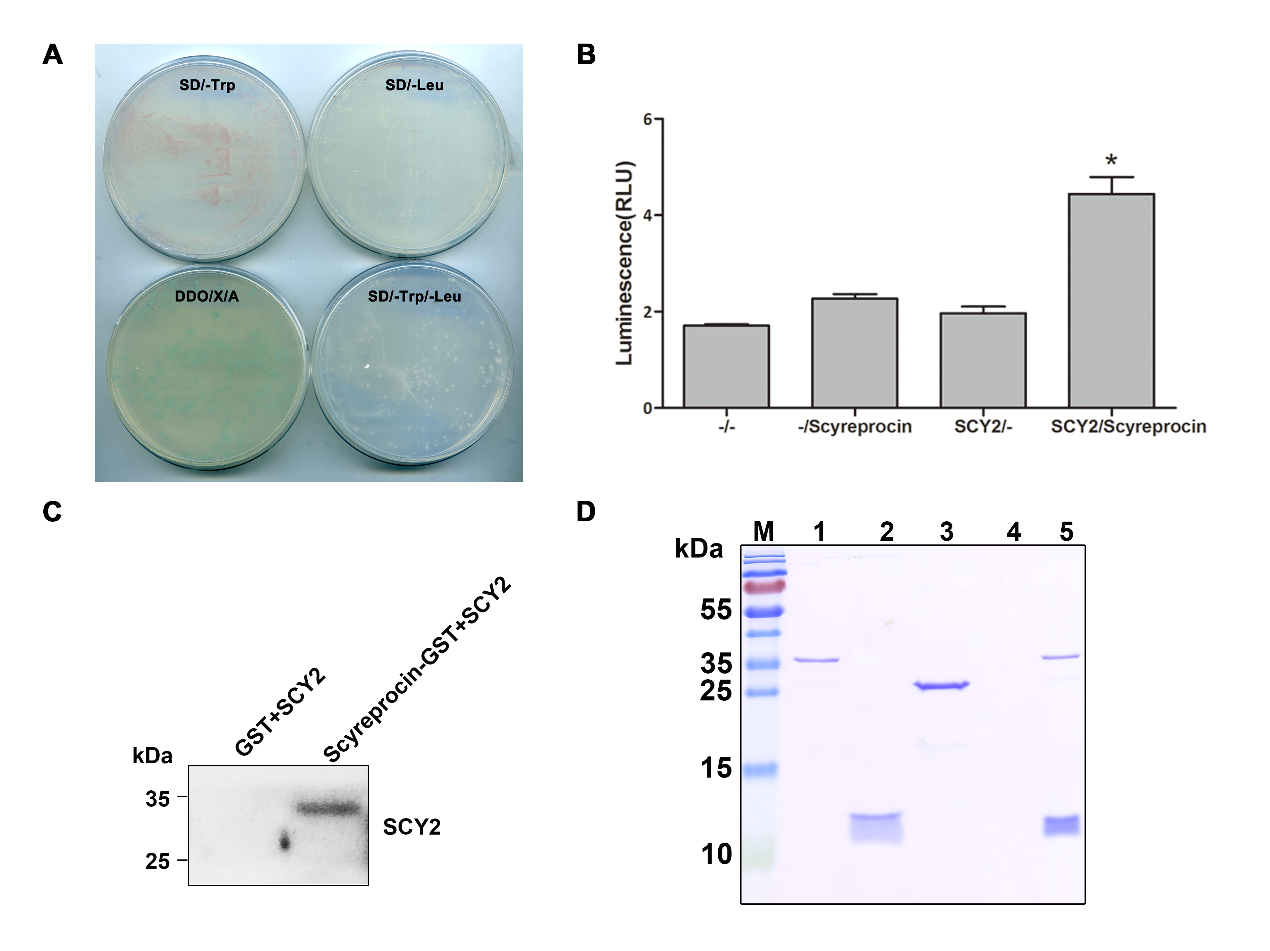


**FIGURE S1 Verification of the interaction between SCY2 and scyreprocin.**

(A) Verification of the scyreprocin-SCY2 interaction by a yeast two-hybrid assay (Y2H) (blue colonies on DDO/X/A plate indicated positive interaction results).

(B) Verification of the scyreprocin-SCY2 interaction by mammalian two-hybrid (M2H) assay. Data represent the means ± SEMs from three independent experiments. *, *P* < 0.05 (one-way ANOVA, repeated measurement).

(C) Verification of the scyreprocin-SCY2 interaction by far-overlay Western blotting. Recombinant SCY2 (rSCY2) was incubated with a membrane blotted with GST and GST-tagged scyreprocin (GST-scyreprocin). The rSCY2 bound on the membrane was detected by SCY2 antibody.

(D) Verification of the scyreprocin-SCY2 interaction by GST-pulldown assay. Samples: 1, GST-scyreprocin; 2, rSCY2; 3, beads + GST + rSCY2; 4, beads + rSCY2; 5, beads + GST-scyreprocin + rSCY2.


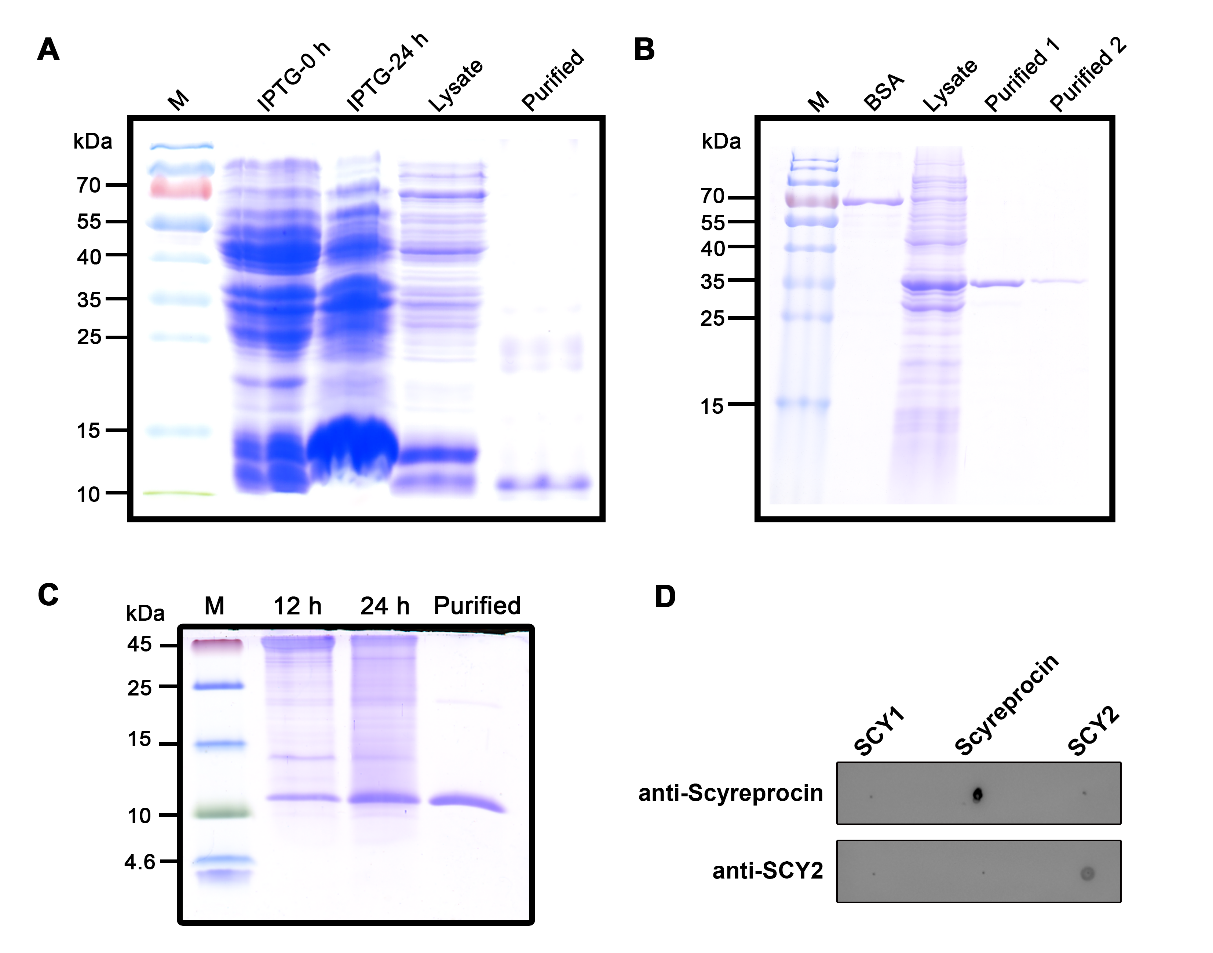


**FIGURE S2 Preparation of recombinant proteins and scyreprocin antibody.**

(A) Expression and purification of rScyreprocin.

(B) Expression and purification of GST-tagged scyreprocin.

(C) Expression and purification of rSCY2.

(D) Antibody specificity test of anti-scyreprocin and anti-SCY2 antibodies.


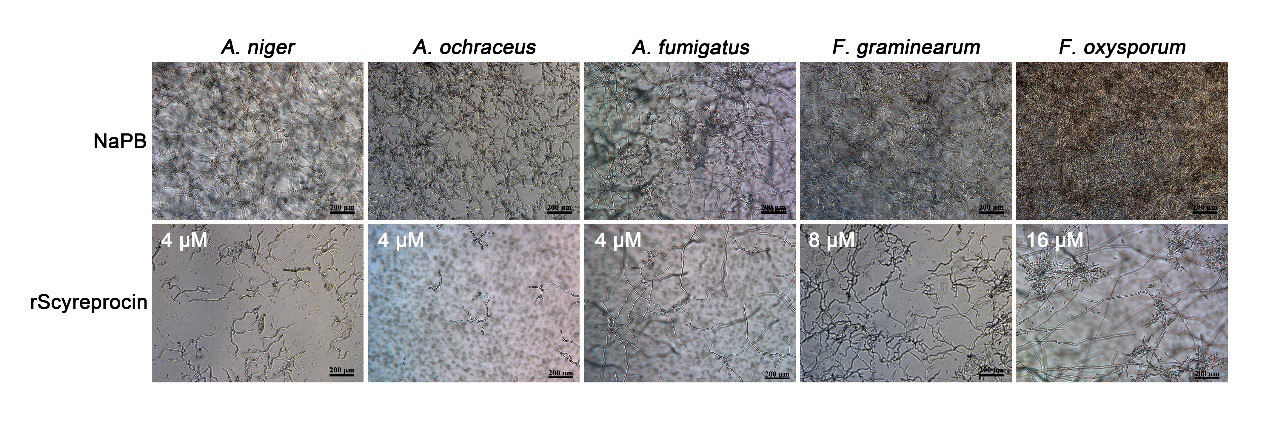


**FIGURE S3 rScyreprocin inhibited spore germination of sporogenous molds.**

Spores of *Aspergillus niger*, *Aspergillus ochraceus*, *Aspergillus fumigatus*, *Fusarium graminearum* and *Fusarium oxysporum* were collected and treated with rScyreprocin or NaPB (control) in growth media at 28℃ for 24 h, samples were then observed by an optical microscope.


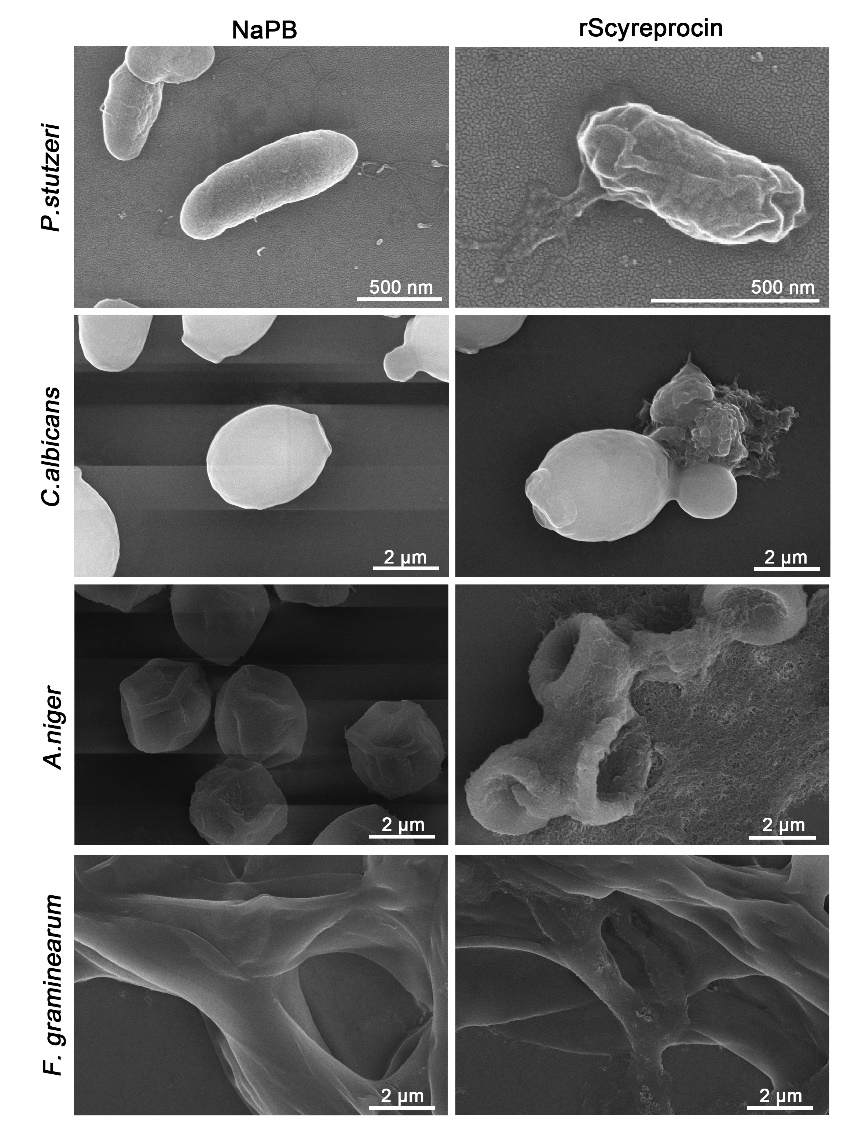


**FIGURE S4 Effect of rScyreprocin on the membrane morphology of *Pseudomonas stutzeri* and *Candida albicans*.**

Exponential phase microbial cells of *P. stutzeri* and *C. albicans* were resuspended in culture media supplemented with NaPB (control) or rScyreprocin (4 μM for *P. stutzeri* and *C. albicans*, 8 μM for *A. niger* spores, 16 μM for *F. graminearum* mycelia) for 30 min and observed using a scanning electron microscopy (SEM). Microbes treated with NaPB (left column) showed a normal smooth surface, while those treated with rScyreprocin (right column) revealed clear morphological changes.


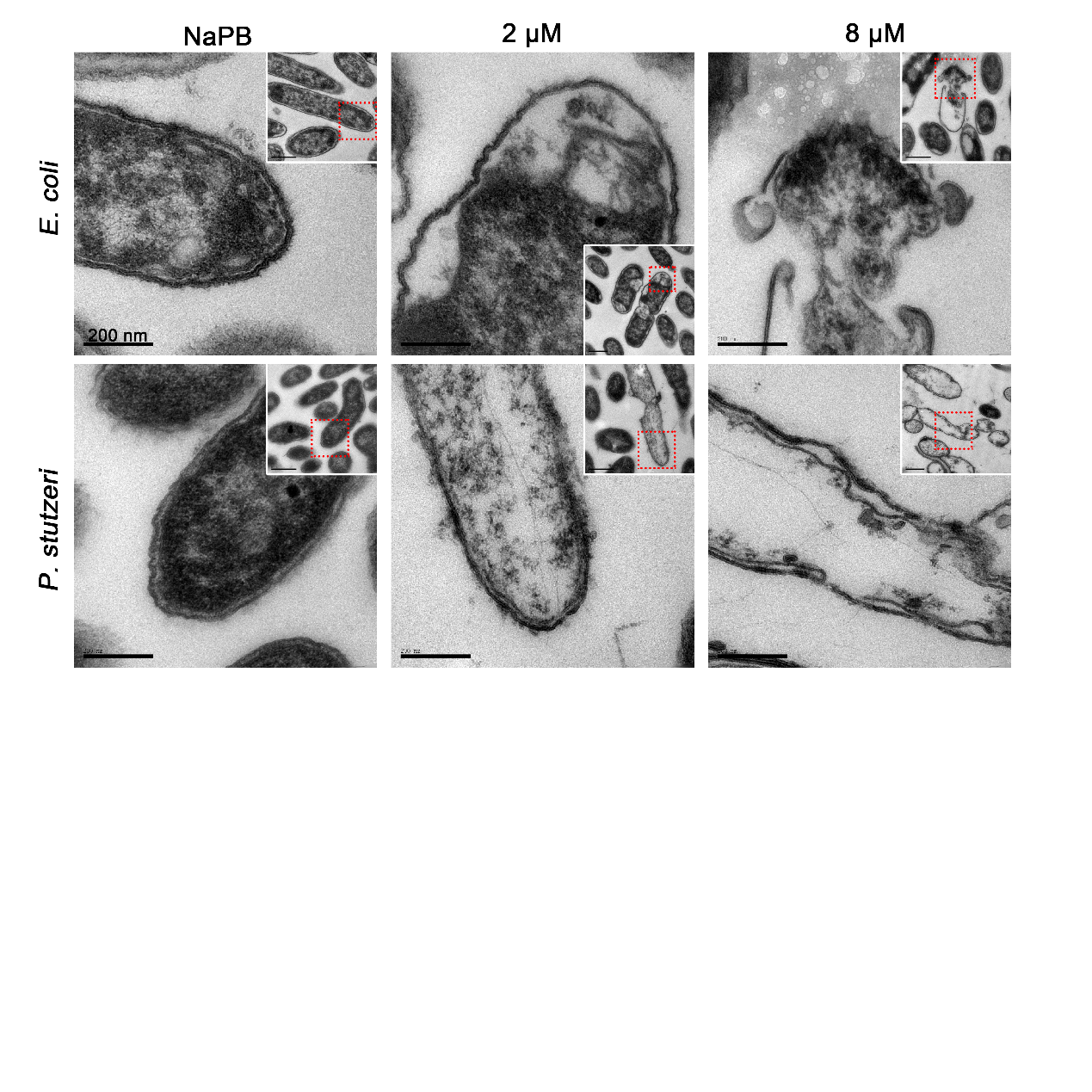


**FIGURE S5 Effect of rScyreprocin on membrane morphology of *Escherichia coli* and *Pseudomonas stutzeri*.**

Exponential phase microbial cells of *E. coli* and *P. stutzeri* were incubated media supplement with rScyreprocin (2 and 8 μM) or NaPB (control) in at 37℃ for 30 min. Samples were observed by a transmission electron microscopy (TEM).


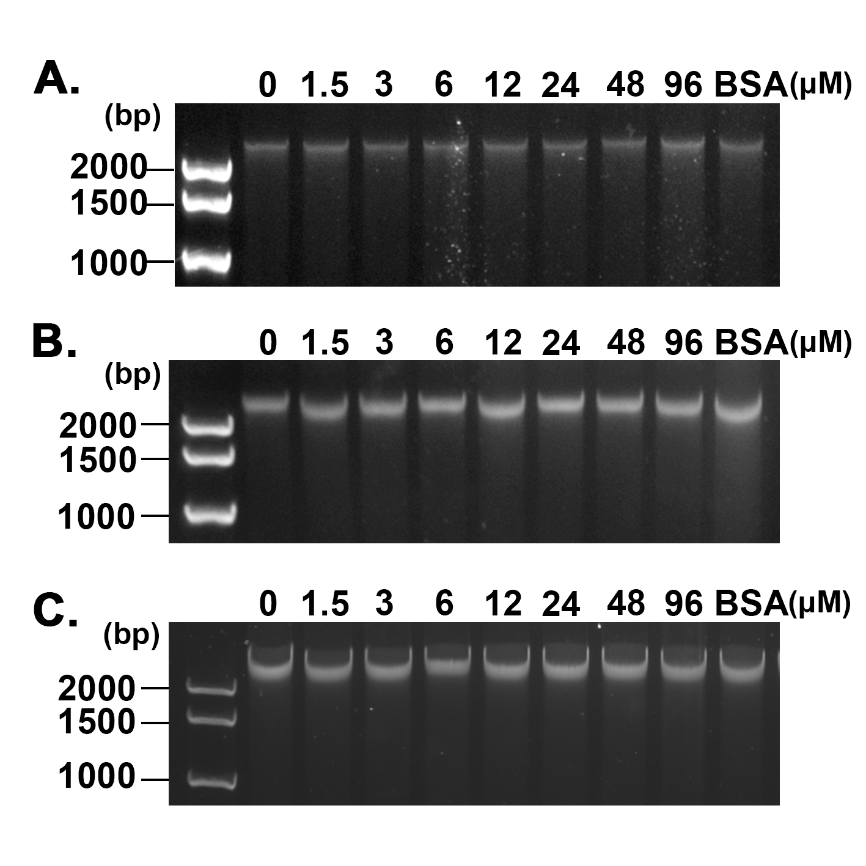


**FIGURE S6 Binding capacity of rScyreprocin with microbial genomic DNA.**

Extracted microbial genomes of *Staphylococcus aureus* (A), *Pseudomonas stutzeri* (B) and *Candida albicans* (C) were incubated with various concentrations of rScyreprocin at room temperature for 30 min. Samples were analyzed by gel electrophoresis.


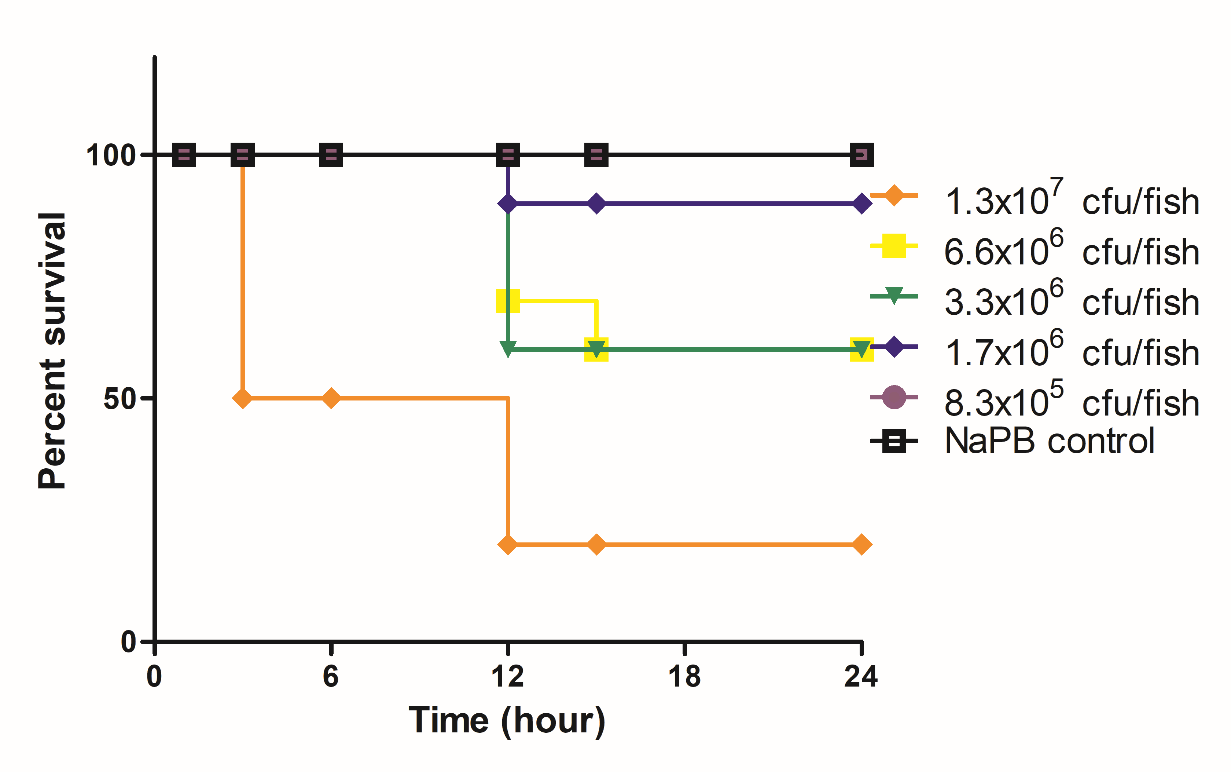


**FIGURE S7 Survival rate of *Vibrio harveyi*-infected *Oryzias melastigma*.**

Marine medaka (*O. melastigma*) was challenged with different amount of *V. harveyi*. Survival rate within 24 h was analyzed using the Kaplan-Meier Log rank test. Data represent the calculated survival rate (n = 20).

**TABLE S1: Primer sequences.**

| **Primer name** | **Forward Primer** | **Reverse Primer** |
| --- | --- | --- |
| ***For Y2H assay*** | | |
| pGBKT7/SCY2 | CATGGAGGCCGAATTCCTCAACAGACTTATGAATAAGGCC | GCAGGTCGACGGATCCTTAGTAGGAAGCAAGCCAGTCCT |
| ***For recombinant expression*** | | |
| pET-28a (+)  /Scyreprocin | CATGCCATGGGAAAGGAAGACAGCAACAT^a^ | CGCTCGAGTTAGTGGTGGTGGTGGTGGTGTTTTACTTTTGGGTCCTT |
| pGEX4T2/Scyreprocin | GCGGGATCCATGAAGGAAGACAGCAAC | CGCTCGAGTTATTTTACTTTTGGGTCCTT |
| ***For M2H assay*** | | |
| SCY2 | CGCGGATCCGGCCTGGCACTCAACAGA | ATTTGCGGCCGCTTAGTAGGAAGCAAGCCA |
| Scyreprocin | CGCGGATCCATGAAGGAAGACAGCAAC | ATTTGCGGCCGCTTATTTTACTTTTGGGTCC |

1. The underlined sequences represent restriction sites.

**TABLE S2: Evaluation of Y2H assay.**

| **Parameters of Y2H assay** | **SCY2** |
| --- | --- |
| Library Titering | 3.17×10^8^ |
| Viability of the Prey library | 1.11×10^8^ |
| Viability of Bait | 1.48×10^5^ |
| Viability of diploids | 5.32×10^4^ |
| Mating Efficiency (%Diploids) | 35.95% |

**TABLE S3: Antimicrobial activity of synthetic scyreprocin segments.**

| **Microorganisms** | **CGMCC No.** | **Scyreprocin[20-39]** | | **Scyreprocin[40-84]** | |
| --- | --- | --- | --- | --- | --- |
|  |  | **MIC (μM)** | **MBC (μM)** | **MIC (μM)** | **MBC (μM)** |
| *Aeromonas hydrophila* | 1.2017 | 6.25-12.5 | >50 | 12.5-25 | >50 |
| *Micrococcus luteus* | 1.634 | >50 | >50 | 12.5-25 | >50 |

**TABLE S4: Quantified fluorescence intensity data of rScyreprocin and PI.**

| **File name** | **Mean Intensity of** |
| --- | --- |
| **Fig 4** | **rScyreprocin** |
| *C. albicans* NaPB | 0.002 |
| *C. albicans* rScyreprocin | 55.433 |
| *P. stutzeri* NaPB | 0.000 |
| *P. stutzeri* rScyreprocin | 60.505 |
| **Fig 5** | **PI** |
| *C. albicans* NaPB | 26.717 |
| *C. albicans* rScyreprocin | 30.370 |
| *P. stutzeri* NaPB | 0.000 |
| *P. stutzeri* rScyreprocin | 83.923 |

**Appendix**

**Appendix 1: Microbial strains information.**

| **CGMCC**  **No.** | **Name** | **Source** |
| --- | --- | --- |
| 1.0634 | *Micrococcus lysodeikticus* | China General Microbiological Culture Collection Center |
| 1.1886 | *Corynebacterium glutamicum* |  |
| 1.2017 | *Aeromonas hydrophila* |  |
| 1.3202 | *Pseudomonas fluorescens* |  |
| 1.376 | *Bacillus cereus* |  |
| 1.2299 | *Micrococcus luteus* |  |
| 1.1593 | *Vibrio harveyi* |  |
| 1.1609 | *Vibrio fluvialis* |  |
| 1.1833 | *Vibrio alginolyticus* |  |
| 1.1615 | *Vibrio parahaemolyticus* |  |
| 1.1803 | *Pseudomonas stutzeri* |  |
| 1.1868 | *Shigella flexneri* |  |
| 1.2389 | *Escherichia coli* |  |
| 1.2421 | *Pseudomonas aeruginosa* |  |
| 1.2465 | *Staphylococcus aureus subsp. aureus* |  |
| 1.3358 | *Bacillus subtilis subsp. subtilis* |  |
| 1.426 | *Staphylococcus epidermidis* |  |
| 1.10753 | *Listeria monocytogenes* |  |
| 2.1563 | *Cryptococcus neoformans* |  |
| 2.2411 | *Candida albicans* |  |
| 2.1975 | *Candida tropicalis* |  |
| 2.1846 | *Candida parapsilosis* |  |
| 2.1857 | *Candida krusei* |  |
| 2.2238 | *Pichia pastoris* |  |
| 3.0316 | *Aspergillus niger* |  |
| 3.441 | *Aspergillus flavus* |  |
| 3.583 | *Aspergillus ochraceus* |  |
| 3.5835 | *Aspergillus fumigatus* |  |
| 3.349 | *Fusarium graminearum* |  |
| 3.584 | *Fusarium solani* |  |
| 3.1604 | *Neurospora crassa* |  |
| 3.6785 | *Fusarium oxysporum* |  |
| - | *Aeromonas sobria* | Fisheries Research Institute of Fujian  Fisheries Research Institute of Fujian |
| - | *Edwardsiella tarda* |  |
| - | *Escherichia coli* MC1061 | Genepower |

**Materials and Methods**

**Peptide synthesis of scyreprocin fragments**

Full-length scyreprocin was synthesized by Bankpeptide, Inc. Hefei, China. The purity of the synthetic peptide determined by HPLC was over 90%. Based on the secondary structure of scyreprocin, the chemically synthesized fragments scyreprocin[1-19], scyreprocin[20-39], scyreprocin[40-84] were produced by GenScripts, Nanjing, China. The purity of the synthetic scyreprocin[1-19] and scyreprocin[40-84] were over 90%, while that of scyreprocin[20-39] was over 85%. The synthetic peptides were stored in -80℃ and dissolved in sterile deionized water prior to use.

**Spore germination inhibition assay.**

Mold strains were revived and plated on appropriate media and incubated until spores were produced. Spores were collected and washed with 50 mM NaPB (pH 7.4) before passing through a cell strainer (100 nm) to remove mycelia. Purified spores were resuspended in potato dextrose water (PDW). Approximately 2.5 × 10^3^ spores well^-1^ were seeded in a 96-well plate, and incubated with various dilutions of rScyreprocin (prepared in deionized water, filter sterilized) for 24 to 48 h at 28℃. Samples were observed by an optical microscope (DMIRB Illuminator, Leica).
